# Accumulation of TIP2;2 Aquaporin during Dark Adaptation Is Partially PhyA Dependent in Roots of *Arabidopsis* Seedlings

**DOI:** 10.3390/plants3010177

**Published:** 2014-03-05

**Authors:** Yumi Uenishi, Yukari Nakabayashi, Ayako Tsuchihira, Mari Takusagawa, Kayo Hashimoto, Masayoshi Maeshima, Kumi Sato-Nara

**Affiliations:** 1 Graduate School of Humanities and Sciences, Nara Women’s University, Nara 630-8506, Japan; E-Mail: mak_hashimoto@cc.nara-wu.ac.jp; 2 Faculty of Science, Nara Women’s University, Nara 630-8506, Japan; 3 Graduate School of Bioagricultural Sciences, Nagoya University, Nagoya 464-8601, Japan; E-Mails: tsuchihira.ayako@f.mbox.nagoya-u.ac.jp (A.T.); maeshima@agr.nagoya-u.ac.jp (M.M.)

**Keywords:** *Arabidopsis thaliana*, aquaporin, dark adaptation, endodermis, light regulation, phytochrome, root, water transport

## Abstract

Light regulates the expression and function of aquaporins, which are involved in water and solute transport. In *Arabidopsis thaliana*, mRNA levels of one of the aquaporin genes, *TIP2;2*, increase during dark adaptation and decrease under far-red light illumination, but the effects of light at the protein level and on the mechanism of light regulation remain unknown. Numerous studies have described the light regulation of aquaporin genes, but none have identified the regulatory mechanisms behind this regulation via specific photoreceptor signaling. In this paper, we focus on the role of phytochrome A (phyA) signaling in the regulation of the TIP2;2 protein. We generated *Arabidopsis* transgenic plants expressing a TIP2;2-GFP fusion protein driven by its own promoter, and showed several differences in TIP2;2 behavior between wild type and the *phyA* mutant. Fluorescence of TIP2;2-GFP protein in the endodermis of roots in the wild-type seedlings increased during dark adaptation, but not in the *phyA* mutant. The amount of the TIP2;2-GFP protein in wild-type seedlings decreased rapidly under far-red light illumination, and a delay in reduction of TIP2;2-GFP was observed in the *phyA* mutant. Our results imply that phyA, cooperating with other photoreceptors, modulates the level of TIP2;2 in *Arabidopsis* roots.

## 1. Introduction

As a consequence of water absorption during development, vacuoles occupy more than 90% of the total volume of plant cells [1]. Vacuoles play multifaceted roles, including accumulation of useful substances, recycling of cell components, regulation of turgor pressure, sequestration of toxic ions, and detoxification of xenobiotics [2,3]. Furthermore, they have a space-filling function. Vacuolar transporters play essential roles in these functions [4]. One group of aquaporins, tonoplast intrinsic proteins (TIPs), serves as channels to pass water and small neutral molecules through the tonoplast [5,6,7]. TIP (TP25 in *Phaseolus vulgaris*) was the first aquaporin reported in plants [8]. In *Arabidopsis thaliana*, ten TIP members are classified into five subgroups, TIP1 to TIP5: these include three TIP1s, three TIP2s, two TIP3s, one TIP4;1 and one TIP5;1 [9]. In addition to water [10,11,12,13,14,15], TIPs are permeable to ammonia [16,17], urea [18], H_2_O_2_ [19] and glycerol [20]. For example, although both TIP1;1 and TIP2;1 are permeable to water [15] and urea [18], TIP1;1 is also permeable to H_2_O_2_ [19] and TIP2;1 is permeable to ammonia [16]. Therefore, each TIP member appears to allow permeation of specific substrate molecules and to play a specific physiological role. The diversity of physiological roles of TIPs is supported by earlier observations that different types of vacuoles contain distinct members of individual TIPs [21,22]. The tonoplast of storage vacuoles contains δ-TIP (presently TIP2), while the lytic vacuoles mainly contain γ-TIP (TIP1) [21]. Three types of TIPs, α-TIP (TIP3), TIP2, and sometimes TIP1, coexist in protein storage vacuoles of seeds [21]. The coexistence and distinct patterns of tissue- and organ-specific expression of TIP members have also been observed in roots and leaves of *Arabidopsis* using fluorescent reporters [23,24,25]. Thus, the expression of TIP isoforms is controlled by developmental cues associated with a specific vacuolar differentiation pattern [26].

External cues such as light and environmental stresses also regulate the expression of *TIPs* [14,27,28,29,30,31]. The expression of several *TIP2* genes is affected by light: *SunTIP7 and SunTIP20* in *Helianthus* leaves are up-regulated by light [28], while expression of others such as *DIP* in *Antirrhinum* cotyledons [32] and *TIP2;2* in *Arabidopsis* roots [31] is increased during the dark period. The modulation of *TIP2s*’ expression by light is probably involved in light regulation of vacuolar functions. However, the mechanism of light regulation of TIP2 and its relationship with vacuolar functions have not been studied with respect to aspects of developmental regulation and stress responses.

The modification of vacuolar functions by increased levels of specific TIPs promotes water permeability in cells and growth of cells and organs. When tomato *SlTIP2;2* is transiently expressed in mesophyll cells in *Arabidopsis*, water permeability of the protoplasts increases [33]. Over-expression of *Panax ginseng PgTIP1* in *Arabidopsis* plants promotes the growth of leaves, roots and seeds [34]. Overexpression of *NtTIP1;1* promotes regeneration, enlargement, and division of tobacco BY-2 cells [35]. Therefore, modulation of *TIP* expression during adaptation to light conditions is probably connected with regulation of water transport and growth in plants through the regulation of vacuolar functions.

Plants monitor light in the environment via multiple photoreceptors such as phytochromes, cryptochromes and phototropins [36,37]. The phytochrome (phy) family encodes red/far-red (FR) light photoreceptors, and *Arabidopsis* has five members, phyA through phyE [38]. Phytochromes generally mediate reversible red/FR light responses [36,39,40]. Furthermore, phyA mediates the very-low-fluence response to broad-spectrum light as a highly sensitive light “antenna” [40,41] as well as the high-irradiation response to FR light [42]. phyA is involved in the regulation of growth and development of not only aerial parts [39] but also roots, affecting root elongation [43], root hair formation [44], and red light-induced root phototropism [45]. One of our research interests is why and how light regulates the growth and development of roots underground.

Our previous study focusing on phyA regulation of gene expression in roots indicated that transcripts of several aquaporin genes including *TIP1;1*, *TIP1;2* and *TIP2;2* increase during dark adaptation and that phyA is involved in regulation of these genes [31]. One of the *TIP* genes strongly expressed in the roots [46], *TIP2;2*, was greatly upregulated during dark adaptation [31]. Light regulation of *TIP2;2* expression could affect the growth and metabolism of roots as a consequence of modification of vacuolar function. However, the effects of light and phyA signaling on TIP2;2 protein levels have not been studied. In this paper, we focused on the roles of light and phyA signaling on the regulation of TIP2;2 protein. We generated transgenic *Arabidopsis* plants expressing a TIP2;2-GFP fusion protein driven by its own promoter, and compared tissue and cellular localization of TIP2;2-GFP in wild-type and *phyA* mutant cells. We also analyzed the protein levels in response to dark conditions and FR illumination and found several differences in gene expression and protein accumulation of TIP2;2 between wild-type and *phyA* mutant cells.

## 2. Results and Discussion

### 2.1. Effects of phyA Mutation on Fluorescent Intensity of TIP2;2-GFP under Light Conditions

In order to examine differences in protein levels of TIP2;2 under light and dark conditions, and between wild-type and *phyA* mutant, we generated transgenic *Arabidopsis* expressing a *TIP2;2-GFP* fusion gene driven by its own promoter (Supplementary Figure S1). RT-PCR and immunoblotting with anti-GFP antibodies confirmed that the TIP2;2-GFP fusion protein is expressed in the transgenic lines (Supplementary Figure S1). Using a confocal laser scanning microscope (LSM), GFP fluorescence was mainly observed on the tonoplast not the plasma membrane, in root cells of all lines (Supplementary Figure S1). Therefore, the exogenous TIP2;2-GFP might be correctly localized in the tonoplast in all transgenic lines. Approximately 1.9 kb of the 5' upstream region, which was used as the *TIP2;2* promoter, contained 521 *cis*-elements based on searching an open database of *cis*-elements, PLACE (plant *cis*-acting regulatory DNA elements) [47]. Of these, 65 *cis*-elements are involved in light regulation (Supplementary Table S1), including the G-box, to which phytochrome interacting factors bind [48], and the conserved I-box sequence found upstream of many light-regulated genes [49]. Other *cis*-elements contained in the sequence were nine sugar-repressive elements [50], eight ABA-responsive elements [51] and 14 auxin-responsive elements [52] (Supplementary Table S1).

#### 2.1.1. Tissue-Specific Localization of TIP2;2-GFP in the Wild Type and *phyA* Mutant

**Figure 1:**
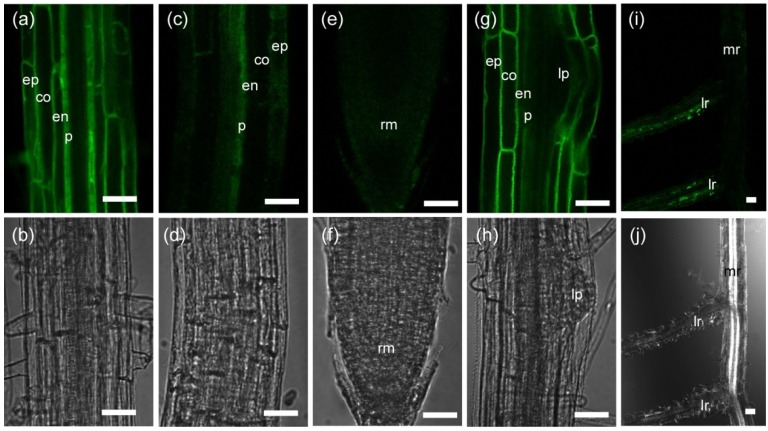
Tissue-specific expression of TIP2;2-GFP in roots of *Arabidopsis* seedlings. Fluorescence (**a**,**c**,**e**,**g**,**i**) and transmitted light (**b**,**d**,**f**,**h**,**j**) images of roots of transgenic Col plants expressing a TIP2;2-GFP fusion protein driven by its own promoter. Seedlings were grown for 6 days (**a**–**h**) or 11 days (**i**,**j**) in a 16 h light/8 h dark cycle. (**a**–**h**) Mature region (**a**,**b**), border region of the elongation to mature zones (**c**,**d**), root tip (**e**,**f**), and lateral root primordium (**g**,**h**) of the primary root. (**i**,**j**) Growing lateral roots with bright fluorescence. co, cortical cell; en, endodermal cell; ep, epidermal cell; lp, lateral root primordium; lr, lateral root; mr, main root; p, pericycle cell; rm, root apical meristem. Bars, 50 µm.

First, to examine differences between two ecotypes, Col and L*er*, and between the wild type (L*er*) and *phyA* mutant, we analyzed the tissue specificity of TIP2;2-GFP expression in roots of light-grown seedlings of Col, L*er* and *phyA* lines. TIP2;2-GFP was detected in the pericycle, endodermis, cortex and epidermis of the mature region of the roots, but not in immature tissues such as the root apical meristem or the developing lateral root primordium (Figure 1).

The strongest fluorescence was detected in the mature region near the differentiation zone, and fluorescence weakened at a more basal region near the hypocotyl. Furthermore, a region of the growing lateral roots in the light-grown plants showed strong fluorescence (Figure 1i). Similar patterns were observed in the roots of all transgenic lines (3 Col, 3 L*er*, and 4 *phyA* background-lines) except for slight differences in the timing of when the tissues began expression and in the fluorescent intensity of the inner tissues, as described below. In Col seedlings, TIP2;2-GFP was first expressed in the pericycle of the border region of the elongation to mature zones and expression expanded to the outer region (Figure 1c). However, in the seedlings with L*er* and *phyA* backgrounds, TIP2;2-GFP was first expressed in the epidermis of the elongation zone (data not shown). This expression pattern resembled that in a previous study reporting that TIP2;2-YFP is first expressed in the cortex and epidermis and then expands to the pericycle during development [23]; these observations of *TIP2;2-YFP* transgenic plants in the Col background differ from ours in that TIP2;2-GFP expression in Col roots started in the pericycle and expanded to the epidermis. This difference may be due to the length of the promoter: Gattolin *et al*. [23] used approximately 1.4 kb of the upstream region of the *TIP2;2* gene as its own promoter, and we used about 1.9 kb. Based on a PLACE database search [47], there are about 160 possible *cis*-elements (Supplementary Table S1), including some involved in light and hormone regulation, in the approximately 550 bp of the upstream region that we included and Gattolin *et al*. [23] did not. Furthermore, based on the differing expression patterns of TIP2;2-GFP in the L*er* and Col backgrounds, the upstream region we used may contain some elements differing between ecotypes. However, additional experiments are necessary to clarify the cause of different tissue-specific localization of the fusion protein between ecotypes, or in our TIP2;2-GFP lines and their TIP2;2-YFP lines.

#### 2.1.2. Fluorescence of TIP2;2-GFP in Light-Grown Wild-Type and *phyA* Mutant Plants

**Figure 2:**
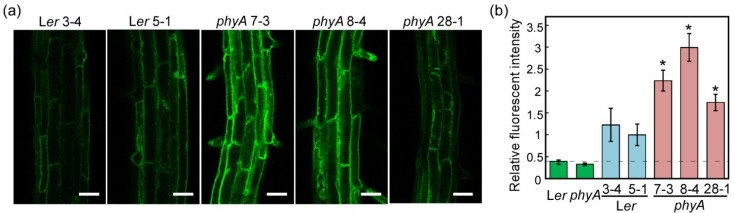
Relative fluorescent intensity in the roots of transgenic *Arabidopsis* expressing a *TIP2;2-GFP* fusion gene in plants of wild-type (L*er*) and *phyA* mutant background. (**a**) Differing fluorescent intensity of TIP2;2-GFP in the roots of light-grown L*er* and *phyA* mutant plants. Plants were grown on MS-agar medium for seven days in a 16 h light/8 h dark cycle. All images were acquired at approximately 5 mm from the root tip by LSM under the same conditions on the same day. Brightness and contrast of images were not modified. Bars, 50 µm; (**b**) Relative fluorescent intensity in the roots of light-grown L*er* and *phyA* seedlings expressing a *TIP2;2-GFP* fusion gene. Relative fluorescent intensity is shown as the fold difference relative to the mean intensity of the L*er*5-1 line. Results are means ± SD of five independent plants. Asterisks indicate statistical differences when compared with L*er*5-1 using Welch’s *t*-test (* *p* < 0.05).

**Figure 3:**
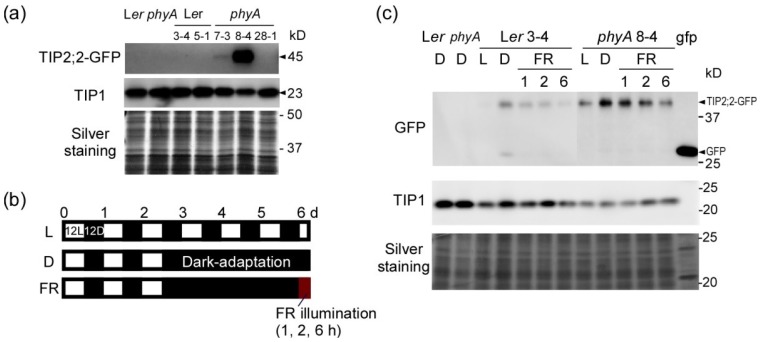
Light-dependent changes in aquaporin abundance in roots of L*er* seedlings and *phyA* mutants during dark adaptation and FR illumination. Aliquots of solubilized crude membrane proteins were immunoblotted with anti-GFP and anti-γ-VM23 (TIP1) antibodies. After detection of TIP2;2-GFP, the same blot was used for TIP1 detection. The background lines were L*er* and *phyA*, and transgenic plants are identified by line number (e.g., L*er*3-4). (**a**) Immunoblots of TIP2;2-GFP in the roots of 7-day light-grown seedlings; (**b**) Diagrams of growth conditions. Seedlings were grown under 12 h irradiation with white light for three days and were then grown continuously in the light (12L12D, L) or dark (D) for another three days. Dark-adapted seedlings were illuminated by FR light for 1, 2 and 6 h; (**c**) Immunoblots of TIP2;2-GFP during dark adaptation and FR illumination. Growth conditions are shown in (**b**).

**Figure 4:**
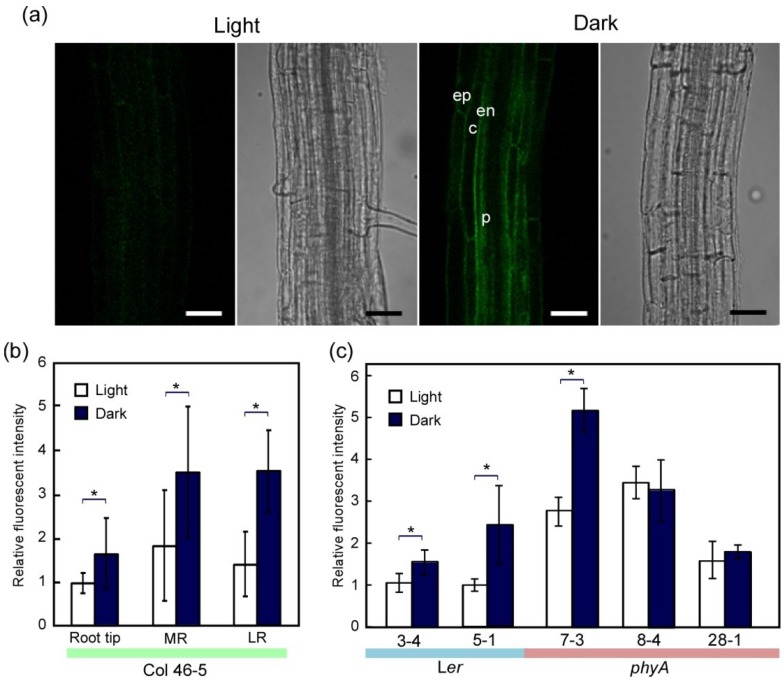
Increase in TIP2;2-GFP protein levels during dark adaptation. (**a**) Fluorescent images of TIP2;2-GFP in the roots of light- and dark-grown TIP2;2-GFP/Col46-5 plants. Seedlings grown for seven days in the light were then grown continuously in the light or dark for another four days. Black and white bars, 50 µm; (**b**) Relative fluorescent intensity of TIP2;2-GFP in the main roots and lateral roots of light- and dark-grown TIP2;2-GFP/Col46-5 plants. The main roots and lateral roots of seven plants were scanned and the brightness of the GFP fluorescence was quantified. Average values for each root were plotted. Scanned areas: Root tip, area approximately 0.5 to 1.2 mm from the root tip of the main root; MR, area approximately 5 to 10 mm from the root tip of the main root; LR, area approximately 0.4 to 1 mm from the root tip of the young lateral root (approximately 0.6 to 1.2 mm long). Asterisks indicate statistical differences found using the Mann-Whitney U-test (* *p* < 0.05); (**c**) Comparison of TIP2;2-GFP signals in the roots of light- and dark-grown TIP2;2-GFP transgenic plants with a L*er* or *phyA* background. Seedlings grown for four days in the light were then grown continuously in the light or dark for three days. The central regions, which contain the central cylinder and protoxylem, of main roots were scanned at approximately 5 mm from the root tip and the brightness of the GFP fluorescence was quantified. Results are means ± SD of seven independent plants. Asterisks indicate statistical differences found using Student’s *t*-test (* *p* < 0.05).

Next, we examined the effects of the *phyA* mutation on TIP2;2-GFP expression. In the roots of light-grown seedlings, the fluorescent intensity of three TIP2;2-GFP/*phyA* lines was higher than that of two TIP2;2-GFP/L*er* lines (Figure 2). This difference in fluorescent intensity was consistent with immunoblotting results (Figure 3a) and other experiments of fluorescent intensity (Figure 4).

We randomly selected *TIP2;2-GFP* transgenic lines in the L*er* and *phyA* backgrounds, but *phyA* lines showed a tendency to be brighter than L*er* lines. To evaluate positional effects due to the insertion region of the transgene, we crossed the brightest line, TIP2;2-GFP/*phyA*8-4, with wild type (L*er*) and observed the fluorescent intensity of the progeny. The segregation ratio of the F_2_ lines was *PHYA*: *phyA* = 3:1 (Table 1, χ^2^ = 0.00456) and *phyA tip2;2-gfp*^−^: other phenotypes = 1:15 (χ^2^ = 1.54). The segregation ratio of the F_2_ lines obtained by backcrossing TIP2;2-GFP/*phyA*8-4 to *phyA-201* was *phyA tip2;2-gfp*^−^: *phyA TIP2;2-GFP*^+^ = 1:3 (χ^2^ = 1.75). Therefore, insertion of the *TIP2;2-GFP* transgene in TIP2;2-GFP/*phyA*8-4 can be considered a single locus unlinked to the *phyA* locus. Under stereoscopic observation, all of the plants with intense green fluorescence had the *phyA* phenotype. More than 60% of the F_2_ progeny with the wild-type phenotype displayed clear or detectable green fluorescence. We selected homozygote lines of *TIP2;2-GFP* and *PHYA* from the F_3_ progeny, and named one of the lines TIP2;2-GFP/L*er*8-4.

**Table 1:** Segregation ratios of wild-type and *phyA* mutated F_2_ progeny from TIP2;2-GFP/*phyA*8-4 plants crossed with L*er* plants or *phyA-201* plants.

F_2_ plant	Number of plants classified with *phyA* mutation and fluorescent intensity *^1^						χ^2^ values *^2^ Expected frequency
	*PHYA*			*phyA*			
	++	+	−	++	+	−	
TIP2;2-GFP/ *phyA* 8-4 × L*er*	0	37	18	2	14	2	0.00456 [PHYA]: [phyA] = 3:1 1.54 [*PHYA* or *GFP*^+^]: [*phyA gfp^−^*] = 15:1
TIP2;2-GFP/ *phyA* 8-4 ×*phyA*	0	0	0	2	18	3	1.75 [*GFP^+^*]:[*gfp^−^*] = 3:1

*^1^ Fluorescent intensities of roots classified into strong (++), detectable (+) and undetectable (−) under stereoscopic observation; *^2^ All the calculated values of χ^2^ were less than the value for χ^2^_0.95_ = 3.84 with one degree of freedom, suggesting that each expected frequency is acceptable.

**Figure 5:**
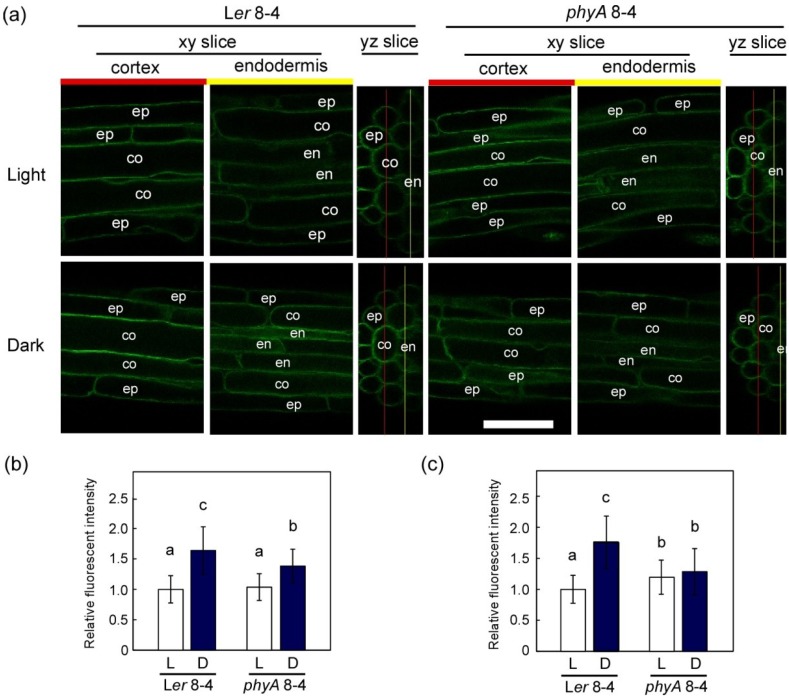
Comparison of TIP2;2-GFP protein levels in epidermal, cortical and endodermal cells between L*er* and *phyA* mutant during dark adaptation. Seedlings grown for three days in the light were then grown continuously in the light or the dark for another three days. The insertion sites of *TIP2;2-GFP* transgenes in the genomes of both L*er*8-4 and *phyA*8-4 lines were identical. (a) Fluorescent images of TIP2;2-GFP in outer (epidermis-cortex) and inner (epidermis-endodermis) optical sections of the roots of light- and dark-grown *TIP2;2-GFP* transgenic plants with a L*er* or *phyA* background. Transverse sections (yz slices) were digitally reconstituted using multiple longitudinal optical sections (xy slices). The red and yellow lines in each yz image indicate the positions of the two xy longitudinal images beside it. The main roots were scanned at approximately 5 mm from the root tip. Bar, 50 µm; (b,c) Comparison of TIP2;2-GFP signals in the roots of light- and dark-grown TIP2;2-GFP transgenic plants with a L*er* or *phyA* background. The brightness of the GFP fluorescence of each image of epidermis-cortex (b) and epidermis-endodermis (c) slices was quantified. Results are means ± SD of three independent plants. Different letters indicate statistical differences found using Scheffe’s F test (*p* < 0.05).

We grew the TIP2;2-GFP/L*er*8-4 and the TIP2;2-GFP/*phyA*8-4 lines in the light and observed them by LSM. In both lines, the fluorescent intensity of TIP2;2-GFP in the outer sliced images containing the epidermis and cortex of the roots was similar (Figure 5a,b). However, in the inner slices containing endodermis of the roots in light-grown plants, the fluorescent intensity of *phyA*8-4 was slightly higher than that of L*er*8-4 (Figure 5a,c). The TIP2;2-GFP fluorescence of the roots of light-grown L*er*8-4 plants tended to be weak in the endodermis, but strong in the epidermis and cortex (Figure 5a). Based on the results, we estimate that the differences in TIP2;2-GFP expression levels between L*er* and *phyA* mutant backgrounds under light conditions (Figure 2, Figure 3 and Figure 4) is largely derived from the differences in the insertion sites of the *TIP2;2-GFP* genes. In the light-grown plants, differences in fluorescent intensity between L*er* and *phyA* are thought to be limited to the inner region of roots (Figure 5).

### 2.2. Dark-Induced Increase in TIP2;2-GFP

Next, we examined the effects of dark adaptation on TIP2;2-GFP expression. In the TIP2;2-GFP/L*er* lines, the amount of TIP2;2-GFP protein increased during dark adaptation (Figure 3). The increase in amounts of TIP2;2-GFP is consistent with the fluorescent intensity of TIP2;2-GFP in the L*er* and Col lines (Figure 4). An increase in protein levels during dark adaptation was also observed in the TIP2;2-GFP/*phyA* lines, although the increase was low in TIP2;2-GFP/*phyA*8-4 (Figure 3). This tendency of each *phyA* line roughly matched the results from image analysis, but the TIP2;2-GFP fluorescence in the center region of *phyA*8-4 roots hardly changed during dark adaptation (Figure 4c). TIP1 also slightly increased during dark adaptation in L*er* but not *phyA* (Figure 3c).

Because the increase in TIP2;2-GFP fluorescence during dark adaptation differed among independent *phyA* lines, we analyzed the TIP2;2-GFP fluorescence of *phyA*8-4 and its progeny L*er*8-4 in detail. In both lines, fluorescent intensity of TIP2;2-GFP in the outer sliced images containing the epidermis and cortex of the roots increased during dark adaptation (Figure 5a,b). The increase in L*er*8-4 was higher than in *phyA*8-4. In the inner slices containing the endodermis of the roots, the fluorescent intensity increased during dark adaptation of L*er*8-4, but not *phyA*8-4 (Figure 5c). Strong TIP2;2-GFP fluorescence in the endodermal cells of dark-adapted L*er*8-4 plants was more frequently observed than in light-grown plants (Figure 5a). Furthermore, in the TIP2;2-GFP/Col lines, stronger fluorescence was observed in the endodermal cells of dark-adapted roots (Figure 4a).

Lack of a dark-adapted increase in TIP2;2-GFP in the endodermal cells of the *phyA* mutant suggests that phyA signaling is involved in the mechanism leading to this change. Although it is not clear why an increase in TIP2;2-GFP in endodermal cells is more phyA-dependent than in the cortex and epidermis, this light response in the endodermal cells might be important for regulating water and solute transport in the roots because the endodermal tissues generally act as an efficient barrier to ions moving through the apoplast [53] and the site where transport of solutes is regulated [54,55].

In the dark-adapted plants, weak GFP fluorescence was also detected in the lumen of the vacuole (Figure 5a). This is consistent with low levels of proteins that had the same size as a GFP monomer (approximately 27 kD) also being detected in the dark-adapted plants (Figure 3c). Conversely, in the light, this presumed GFP monomer was not detected. Vacuolar GFP is rapidly degraded in the light [56], suggesting that tonoplast aquaporins taken up into vacuoles are also degraded at a constant speed in the light, and their degradation is slowed down in the dark. This behavior may lead to a dark-induced increase in the amount of aquaporins.

### 2.3. PhyA Regulation of Aquaporins

PhyA is the sole photoreceptor to respond to continuous FR light in *Arabidopsis* [57]. To confirm phyA regulation of the TIP2;2 protein, we compared the changes in protein levels in the roots of L*er* and a *phyA* mutant under FR illumination. In the roots of the L*er* lines, the amount of TIP2;2-GFP protein decreased 1 h after FR illumination, and further decreased by 6 h (Figure 3c). In *phyA* roots, the amount of TIP2;2-GFP slightly decreased at 1 h after FR illumination, and stayed at relatively high levels at 2 and 6 h after FR illumination. These different patterns of protein levels in L*er* and *phyA* as well as the lack of a dark-adapted increase in TIP2;2-GFP in the *phyA* mutant suggest that phyA signaling is partly involved in the reduction in TIP2;2-GFP in the light. Further studies are required to clarify the direct and indirect roles of phyA in regulation of the TIP2;2 abundance.

Modification of the rates of transcription [31] and translation, as well as degradation, of the protein might be involved in this rapid drop in TIP2;2-GFP 1 h after FR illumination (Figure 3c). In the *phyA* mutant, the observed slower rate of decrease suggests the involvement of phyA in the early phases of FR illumination. In later phases, TIP2;2-GFP levels were similar to those in the light, suggesting that other factors are also involved in this process. The involvement of other factors are also indicated by fluorescent analysis of epidermal and cortical cells, in which the *phyA* mutant showed a dark-induced increase in TIP2;2-GFP (Figure 5b) and by immunoblots using crude membrane proteins from whole roots (Figure 3c). The results indicate that a light-responsive mechanism to decrease the amount of TIP2;2 functions normally in wild-type plants, but this function is weakened in the *phyA* mutant. In the vacuolar lumen, all proteins seem to be more rapidly degraded in the light than in the dark [56]. It is not known how tonoplast proteins are degraded, or whether the degradation mechanisms of both the proteins in the vacuolar lumen and in the tonoplast are similar. Further analysis of the mechanisms behind the sudden drop in TIP2;2-GFP in the early phase of FR exposure may provide insight into tonoplast protein degradation systems in both the dark and light.

### 2.4. Why TIP2;2 Increases during Dark Adaptation

One of the mechanisms responsible for the increase in amount of TIP2;2 protein in the dark is transcriptional regulation of the gene. In addition to numerous *cis*-elements in light regulation, multiple potential sugar-repressive elements [50] are included in the *TIP2;2* promoter (Supplementary Table S1). Furthermore, the *TIP2;2* gene is induced by decreasing the sucrose concentration in the medium [31]. Thus, decreasing the amount of sucrose or photosynthetic products during a long dark period could cause an increase in aquaporin gene expression. Aquaporins can transport small neutral molecules as well as water [6]. It is thought that limited energy in the dark and degradation of various macromolecules [58] brings about the necessity of transporting toxic ammonia/ammonium either to the vacuole for isolation or to other subcellular compartments for production of other dark-required compounds. With respect to the pKa of 9.24 for the deprotonation of ammonium to ammonia and the cytosolic pH of 7.0 to 7.5, approximately 1% of the total cytoplasmic ammonia/ammonium is present in an uncharged form [16]. The uncharged form of ammonia can be passively transported by TIP2 subfamily of aquaporins, TIP2;1 and TIP2;3, from the cytosol to the vacuole [16]. The ammonia diffusing across the tonoplast subsequently binds a proton to form ammonium, which is trapped in the acidic vacuolar lumen.

TIP2;2 is quite similar to TIP2;1 and TIP2;3, which transport water, urea, and ammonia [15,16,18], thus suggesting that TIP2;2 also transports these molecules. The increase in the amount of TIP2;2-GFP during dark adaptation may compensate for the lowering transport efficiency of aquaporins. Increased aquaporins might facilitate efficient water and solute transport across the membranes, even if the driving forces such as osmotic and hydrostatic pressure gradients are lowered. The passive transport of ammonia by aquaporins is dependent on the pH gradient between the cytosol and the vacuolar lumen, which is mainly generated by two proton pumps [4]. The long dark condition without photosynthesis could lower the activities of proton pumps, and thus lower the pH-dependent transport of ammonia by aquaporins as well as other transporters whose transport is dependent on a chemical and/or electrical gradient between both sides of the tonoplast. Water transport by aquaporins is also correlated with solute transport because the osmotic pressure gradient is one of the driving forces of water movement [59].

Vacuoles can be classified by the type of TIP found in the tonoplast and TIP2;2 is a type of δ-TIP, which is generally in the tonoplast of storage vacuoles [4]. Because TIP2;2 is also one of the major aquaporins in *Arabidopsis* roots [46], it probably plays an important role in light-grown plants. The increase in TIP2;2 during dark adaptation suggests that vacuolar function is modified by light and that the physiological function of TIP2;2-containing vacuoles may be altered during long dark periods.

Alternatively, TIP2;2, in concert with other aquaporins, may facilitate water flow from the epidermis to the xylem in the central cylinder of roots. Intriguingly, TIP2;2-YFP and TIP2;3-YFP are present in the rows of pericycle cells that form the xylem poles [23]. We did not examine the fluorescent intensity of pericycle cells or the location of fluorescent cells, although several pericycle cells displayed clear fluorescence (Figure 4 and Figure 5). The increase in TIP2;2 in the endodermal cells during dark adaptation is also associated with the important role of transport of water and solutes into the central cylinder. Further analysis of the mechanism of light regulation of aquaporins should lead to a better understanding of the tissue- and cell-specific roles of TIP2;2 in both the light and dark.

## 3. Experimental

### 3.1. Plant Material and Growth Conditions

*Arabidopsis thaliana* L*er* (NW20) and *phyA-201* (N6219) lines were obtained from the NASC (Nottingham Arabidopsis Stock Centre, Loughborough, UK), and Col-0 (J58) was obtained from the SASSC (Sendai Arabidopsis Seed Stock Center, Sendai, Japan). Seeds were surface-sterilized and sown on the surface of Murashige and Skoog (MS) medium containing 1% agar and 1% sucrose in square styrene plastic cases (90 × 70 × 23 mm). Approximately 20 seeds (for fluorescent intensity analysis) or 300 seeds (for protein extraction) per case were sown in two rows. After vernalization at 4 °C for two days, seeds were cultured vertically at 22 °C under 12 or 16 h illumination (12L12D or 16L8D photoperiods) with cool white fluorescent light of 15 W/m^2^. FR light was provided by a light-emitting diode (LED; 730 nm, 6.2 W/m^2^; MIL-IF18; Sanyo, Tokyo, Japan) with MIL-U200 frames and a MIL-C1000T controller system. Fluence rates were measured with a model LI-250 radiometer (Li-Cor, Lincoln, NE, USA). Roots of the plants under dark adaptation were harvested using a glove box in total darkness, as described previously [31]. Sampling tubes containing roots were wrapped in aluminum foil before withdrawing them from the glove box, and were immediately frozen in liquid nitrogen. Frozen roots were stored at −80 °C.

### 3.2. Fluorescent Intensity Analysis

GFP fluorescence intensity was measured using a C1siR confocal LSM (Nikon Instruments, Tokyo, Japan). Fresh roots excised from seedlings were immediately immersed in *Arabidopsis* hydroponic medium [60] on slides and were observed by LSM. To image GFP, the 488 nm line was used for excitation, and emission was detected at 515/30 nm. For semiquantitative measurement of fluorescence intensity, the laser, pinhole and gain settings of the LSM were kept identical among treatments. The fluorescence intensity of each digital image was analyzed using EZ-C1 software [61] as shown in Supplementary Figure S2. Images were assembled using Photoshop software [62].

### 3.3. Protein Preparation

Frozen roots (100–200 mg) were crushed with a Multi-beads Shocker cell disruptor (Yasui Kikai, Osaka, Japan), after which 7 µL mg^−1^ root of cold extraction buffer (100 mM Tris-HCl (pH 8.0), 300 mM NaCl, 20 mM EDTA, 20% (*w*/*v*) glycerol, 5 mM dithiothreitol (DTT) and 1× Complete Protease Inhibitor Cocktail (Roche Applied Science, Tokyo, Japan)) was immediately added, and the mixture was homogenized with the Multi-beads Shocker at 4 °C. The homogenate was strained through a filtration apparatus and centrifuged at 10,000 rpm for 10 min at 4 °C. The supernatant was recovered and centrifuged again at 100,000× *g* for 30 min at 4 °C. The pellet was used as the microsomal membrane fraction. The pellet was resuspended in 0.2 µL mg^−1^ root extraction buffer. After protein concentrations were determined, each microsomal membrane fraction was frozen in liquid nitrogen and stored at −80 °C. Protein concentrations of the soluble and membrane protein samples were determined using a Bio-Rad protein assay kit (Bio-Rad, Tokyo, Japan) with γ-globulin as a standard.

### 3.4. Immunoblotting

Each microsomal membrane fraction was mixed with an equal volume of 2× SDS-PAGE sample buffer and incubated for 10 min at 70 °C. SDS-PAGE in 9% and 12% (*w*/*v*) polyacrylamide gels containing 0.1% SDS was carried out by the method of Laemmli (1970). For immunoblotting, proteins in polyacrylamide gels were transferred to a Clear Blot P polyvinylidene difluoride membrane (ATTO, Tokyo, Japan) with a semidry blotting apparatus using standard procedures. The membrane was blocked with 2% (*w*/*v*) ECL Advance Blocking Agent (GE Healthcare, Tokyo, Japan) in TBS-T buffer (20 mM Tris-HCl (pH 7.6), 0.14 M NaCl, 0.1% (*v*/*v*) Tween 20) overnight at 4 °C. The membrane was incubated with the appropriate primary antibody for 1 h at 20 °C. Concentrations of antibodies were: 1:500 dilution of anti-GFP antibody (Clontech A.v. peptide antibody, polyclonal; Takara-Bio, Shiga, Japan) and 1:10,000 dilution of anti-TIP1 antibody (γVM23, [63]). After washing for 15 min and another three times for 5 min with TBS-T, blots were incubated with secondary antibody, a 1:100,000 dilution of ECL anti-rabbit IgG, horseradish peroxidase-linked species specific whole antibody (from donkey, NA934V, GE Healthcare, Tokyo, Japan) for 1 h at 20 °C. After washing as described above, antibody conjugates were detected using an ECL Advance chemiluminescent reagent (GE Healthcare, Tokyo, Japan) according to the manufacturer’s instructions. Chemiluminescence images were acquired using a LAS-3000 luminescent imaging system (Fujifilm, Tokyo, Japan), and were quantitated using MultiGauge software [64]. After detection, each immunoblot was stripped of probe according to Kaufmann and Kellner [65], and reprobed using an anti-TIP1 antibody. All protein samples used for immunoblotting were electrophoresed on other gels and gels were stained by standard procedures with silver or Coomassie brilliant blue R-250.

### 3.5. Generation of TIP2;2-GFP Transgenic Lines

In order to generate a TIP2;2 promoter::TIP2;2::sGFP construct (TIP2;2-GFP), a 2.9-kb genomic region containing the *TIP2;2* gene and a 1.9 kb upstream region was PCR amplified from the genomic DNA of Col-0 using primers TIP2;2-1190F and TIP2;2-4035R (Supplementary Table S2). PCR products were cloned into vector ENTR/D-TOPO (Invitrogen, Tokyo, Japan) before recombination into the destination vector pGWB404 using Gateway technology [66]. Correct in-frame GFP insertion was verified by DNA sequencing. The construct was introduced into *Agrobacterium tumefaciens* GV3101 before transformation of Arabidopsis Col-0, L*er*, and *phyA-201* plants by floral dipping [67]. Transgenic plants were either selected on kanamycin or identified by GFP fluorescence of roots under an M165 FC fluorescence stereomicroscope (Leica Microsystems, Tokyo, Japan). From 15,000 seeds of Col and 5000 seeds each of L*er* and *phyA*, 119 Col, 24 L*er* and 30 *phyA* kanamycin-resistant T_1_ plants were selected. More than 10 independent T_2_ plants were further selected. The transgenic lines showing 100% kanamycin resistance were observed under a confocal LSM. F_1_, F_2_, and F_3_ progeny of TIP2;2-GFP/*phyA* 8-4 crossed to L*er* were selected by the hypocotyl phenotype of the seedlings grown under FR illumination for five days [68] in addition to GFP fluorescence and kanamycin resistance.

### 3.6. Protoplast Preparation

Roots of six-day-old seedlings (Col-0, TIP2;2-GFP-46-5 and TIP2;2-GFP-40) were cut longitudinally with a razor while immersed in a few drops of Arabidopsis hydroponic medium (0.015 M NaH_2_PO_4_, 2.6 mM Na_2_HPO_4_, 1.5 mM MgSO_4_, 0.03 M KNO_3_, 0.14 mM Fe(III) EDTA, 0.02 M Ca(NO_3_)_2_, 0.1 mM MnSO_4_, 0.3 mM H_3_BO_3_, 0.01 mM ZnSO_4_, 9.6 µM CuSO_4_, 0.24 µM (NH_4_)_6_Mo_7_O_24_, 1.2 µM CoC1_2_ [60]) on a slide. Root tissues were transferred to a sample tube, and were incubated in 0.2 mL protoplast enzyme solution [2% (*w*/*v*) cellulase Onozuka RS (Yakult Pharmaceutical, Tokyo, Japan), 0.1% (*w*/*v*) macerozyme R-10 (Yakult Pharmaceutical, Tokyo, Japan), 0.03% (*w*/*v*) pectolyase Y23 (Kikkoman, Tokyo, Japan), 20 mM MES-KOH (pH 5.5), 2 mM DTT, 0.39 M sorbitol] at 22 °C for 60 to 90 min [69]. Enzyme solution was then gently removed and protoplasts were washed twice in 0.5 mL washing solution (0.4 M sorbitol in Arabidopsis hydroponic medium). Gravitated protoplasts were gently suspended in 20 µL of washing solution and were observed under a confocal LSM.

### 3.7. RT-PCR

Roots of two-month-old plants (Col-0, TIP2;2-GFP-46-5 and TIP2;2-GFP-40) were excised and immediately immersed in Ambion RNAlater reagent (Applied Biosystems, Tokyo, Japan). The immersed roots were kept at 4 °C overnight. Fixed tissues were removed from the reagent, immediately frozen in liquid nitrogen, and stored at −80 °C. Frozen samples were crushed using the Multi-beads Shocker and total RNA was isolated using an RNeasy Plant Mini Kit (Qiagen, Tokyo, Japan). After treatment with RNase-free DNase I (Invitrogen, Tokyo, Japan), a 1 µg aliquot of total RNA was reverse transcribed using 200 U PrimeScript II RTase (Takara Bio, Otsu, Japan). cDNA was amplified by PCR using Ex Taq polymerase (Takara Bio) and primers TIP2;2cDNA-1F and TIP2;2-sGFP-976R (Supplementary Table S2). TIP2;2-sGFP-1266R was also used. PCR of TIP2;2-GFP was performed as follows: 94 °C for 9 min; 32 cycles at 94 °C for 30 s, 57.3 °C for 30 s, and 72 °C for 1 min; then 72 °C for 10 min. Expression levels of Actin-8 (At1g49240) were also examined as an internal control. PCR of Actin-8 was performed under the same conditions as for TIP2;2-GFP except for annealing temperature (55 °C), extension time (30 s), and primers (ACT8F and ACT8R, Supplementary Table S2).

## 4. Conclusions

In this paper, we presented evidence for the involvement of a phyA signaling pathway in light regulation of aquaporin TIP2;2. We also proposed that phyA signaling directly or indirectly influences the tissue-specific localization of TIP2;2-GFP and/or functional modification of the tonoplast. These modifications to the amount and localization of TIP2;2 during dark adaptation and FR illumination could change the permeability to water and some solutes via the tonoplast of root cells. During a long dark period, various slow-acting cellular processes allow survival. TIP2;2 may play a role in such processes in the roots of dark-adapted plants. The findings in this paper provide a basis to clarify the mechanisms regulating water and solute transport based on a plant’s light environment.

## Supplementary Files

Supplementary File 1

## References

[1] Taiz L. (1992). The plant vacuole. J. Exp. Biol..

[2] Marty F. (1999). Plant vacuoles. Plant Cell.

[3] Frigerio L., Hinz G., Robinson D.G. (2008). Multiple vacuoles in plant cells: Rule or exception?. Traffic.

[4] Martinoia E., Maeshima M., Neuhaus H.E. (2007). Vacuolar transporters and their essential role in plant metabolism. J. Exp. Bot..

[5] Maurel C. (2007). Plant aquaporins: Novel functions and regulation properties. FEBS Lett..

[6] Katsuhara M., Hanba Y.T., Shiratake K., Maeshima M. (2008). Review: Expanding roles of plant aquaporins in plasma membranes and cell organelles. Funct. Plant Biol..

[7] Maurel C., Santoni V., Luu D.T., Wudick M.M., Verdoucq L. (2009). The cellular dynamics of plant aquaporin expression and functions. Curr. Opin. Plant Biol..

[8] Johnson K.D., Herman E.M., Chrispeels M.J. (1989). An abundant, highly conserved tonoplast protein in seeds. Plant Physiol..

[9] Johanson U., Karlsson M., Johansson I., Gustavsson S., Sjövall S., Fraysse L., Weig A.R., Kjellbom P. (2001). The complete set of genes encoding major intrinsic proteins in *Arabidopsis* provides a framework for a new nomenclature for major intrinsic proteins in plants. Plant Physiol..

[10] Maurel C., Reizer J., Schroeder J.I., Chrispeels M.J. (1993). The vacuolar membrane protein γ-TIP creates water specific channels in *Xenopus* oocytes. EMBO J..

[11] Kaldenhoff R., Fischer M. (2006). Functional aquaporin diversity in plants. Biochim. Biophys. Acta.

[12] Maurel C., Kado R.T., Guern J., Chrispeels M.J. (1995). Phosphorylation regulates the water channel activity of the seed-specific aquaporin α-TIP. EMBO J..

[13] Leitão L., Prista C., Moura T.F., Loureiro-Dias M.C., Soveral G. (2012). Grapevine aquaporins: Gating of a tonoplast intrinsic protein (TIP2;1) by cytosolic pH. PLoS One.

[14] Vera-Estrella R., Barkla B.J., Bohnert H.J., Pantoja O. (2004). Novel regulation of aquaporins during osmotic stress. Plant Physiol..

[15] Daniels M.J., Chaumont F., Mirkov T.E., Chrispeels M.J. (1996). Characterization of a new vacuolar membrane aquaporin sensitive to mercury at a unique site. Plant Cell.

[16] Loqué D., Ludewig U., Yuan L., von Wirén N. (2005). Tonoplast intrinsic proteins AtTIP2;1 and AtTIP2;3 facilitate NH_3_ transport into the vacuole. Plant Physiol..

[17] Jahn T.P., Møller A.L., Zeuthen T., Holm L.M., Klaerke D.A., Mohsin B., Kühlbrandt W., Schjoerring J.K. (2004). Aquaporin homologues in plants and mammals transport ammonia. FEBS Lett..

[18] Liu L.H., Ludewig U., Gassert B., Frommer W.B., von Wirén N. (2003). Urea transport by nitrogen-regulated tonoplast intrinsic proteins in *Arabidopsis*. Plant Physiol..

[19] Bienert G.P., Moller A.L., Kristiansen K.A., Schulz A., Moller I.M., Schjoerring J.K., Jahn T.P. (2007). Specific aquaporins facilitate the diffusion of hydrogen peroxide across membranes. J. Biol. Chem..

[20] Gerbeau P., Güçlü J., Ripoche P., Maurel C. (1999). Aquaporin Nt-TIPa can account for the high permeability of tobacco cell vacuolar membrane to small neutral solutes. Plant J..

[21] Jauh G.Y., Phillips T.E., Rogers J.C. (1999). Tonoplast intrinsic protein isoforms as markers for vacuolar functions. Plant Cell.

[22] Maeshima M., Hara-Nishimura I., Takeuchi Y., Nishimura M. (1994). Accumulation of vacuolar H^+^-pyrophosphatase and H^+^-ATPase during reformation of the central vacuole in germinating pumpkin seeds. Plant Physiol..

[23] Gattolin S., Sorieul M., Hunter P.R., Khonsari R.H., Frigerio L. (2009). *In vivo* imaging of the tonoplast intrinsic protein family in *Arabidopsis* roots. BMC Plant Biol..

[24] Gattolin S., Sorieul M., Frigerio L. (2011). Mapping of tonoplast intrinsic proteins in maturing and germinating *Arabidopsis* seeds reveals dual localization of embryonic TIPs to the tonoplast and plasma membrane. Mol. Plant.

[25] Hunter P.R., Craddock C.P., di Benedetto S., Roberts L.M., Frigerio L. (2007). Fluorescent reporter proteins for the tonoplast and the vacuolar lumen identify a single vacuolar compartment in *Arabidopsis* cells. Plant Physiol..

[26] Wudick M.M., Luu D.T., Maurel C. (2009). A look inside: Localization patterns and functions of intracellular plant aquaporins. New Phytol..

[27] Suga S., Komatsu S., Maeshima M. (2002). Aquaporin isoforms responsive to salt and water stresses and phytohormones in radish seedlings. Plant Cell Physiol..

[28] Maurel C., Javot H., Lauvergeat V., Gerbeau P., Tournaire C., Santoni V., Heyes J. (2002). Molecular physiology of aquaporins in plants. Int. Rev. Cytol..

[29] Kim H.S., Yu Y., Snesrud E.C., Moy L.P., Linford L.D., Haas B.J., Nierman W.C., Quackenbush J. (2005). Transcriptional divergence of the duplicated oxidative stress-responsive genes in the *Arabidopsis* genome. Plant J..

[30] Liu F., Vantoai T., Moy L.P., Bock G., Linford L.D., Quackenbush J. (2005). Global transcription profiling reveals comprehensive insights into hypoxic response in *Arabidopsis*. Plant Physiol..

[31] Sato-Nara K., Nagasaka A., Yamashita H., Ishida J., Enju A., Seki M., Shinozaki K., Suzuki H. (2004). Identification of genes regulated by dark adaptation and far-red light illumination in roots of *Arabidopsis thaliana*. Plant Cell Environ..

[32] Culianez-Macia F.A., Martin C. (1993). DIP: A member of the MIP family of membrane proteins that is expressed in mature seeds and dark-grown seedlings of *Antirrhinum majus*. Plant J..

[33] Sade N., Vinocur B.J., Diber A., Shatil A., Ronen G., Nissan H., Wallach R., Karchi H., Moshelion M. (2009). Improving plant stress tolerance and yield production: Is the tonoplast aquaporin SlTIP2;2 a key to isohydric to anisohydric conversion?. New Phytol..

[34] Lin W.L., Peng Y.H., Li G.W., Arora R., Tang Z.C., Su W.A., Cai W.M. (2007). Isolation and functional characterization of PgTIP1 a hormone-autotrophic cells-specific tonoplast aquaporin in ginseng. J. Exp. Bot..

[35] Okubo-Kurihara E., Sano T., Higaki T., Kutsuna N., Hasezawa S. (2009). Acceleration of vacuolar regeneration and cell growth by overexpression of an aquaporin NtTIP1;1 in tobacco BY-2 cells. Plant Cell Physiol..

[36] Whitelam G.C., Halliday K.J. (2007). Light and Plant Development.

[37] Van Buskirk E.K., Decker P.V., Chen M. (2012). Photobodies in light signaling. Plant Physiol..

[38] Clack T., Mathews S., Sharrock R.A. (1994). The phytochrome apoprotein family in *Arabidopsis* is encoded by five genes: The sequences and expression of *PHYD* and *PHYE*. Plant Mol. Biol..

[39] Kami C., Lorrain S., Hornitschek P., Fankhauser C. (2010). Light-regulated plant growth and development. Curr. Top. Dev. Biol..

[40] Franklin K.A., Quail P.H. (2010). Phytochrome functions in *Arabidopsis* development. J. Exp. Bot..

[41] Botto J.F., Sánchez R.A., Whitelam G.C., Casal J.J. (1996). Phytochrome A mediates the promotion of seed germination by very low fluences of light and canopy shade light in *Arabidopsis*. Plant Physiol..

[42] Hennig L., Büche C., Schäfer E. (2000). Degradation of phytochrome A and the high irradiance response in *Arabidopsis*: A kinetic analysis. Plant Cell Environ..

[43] Correll M.J., Kiss J.Z. (2005). The roles of phytochromes in elongation and gravitropism of roots. Plant Cell Physiol..

[44] De Simone S., Oka Y., Inoue Y. (2000). Effect of light on root hair formation in *Arabidopsis thaliana* phytochrome-deficient mutants. J. Plant Res..

[45] Kiss J.Z., Mullen J.L., Correll M.J., Hangarter R.P. (2003). Phytochromes A and B mediate red-light-induced positive phototropism in roots. Plant Physiol..

[46] Boursiac Y., Chen S., Luu D.T., Sorieul M., van den Dries N., Maurel C. (2005). Early effects of salinity on water transport in *Arabidopsis* roots. Molecular and cellular features of aquaporin expression. Plant Physiol..

[47] Higo K., Ugawa Y., Iwamoto M., Korenaga T. (1999). Plant *cis*-acting regulatory DNA elements (PLACE) database: 1999. Nucleic Acids Res..

[48] Castillon A., Shen H., Huq E. (2007). Phytochrome Interacting Factors: Central players in phytochrome-mediated light signaling networks. Trends Plant Sci..

[49] Terzaghi W.B., Cashmore A.R. (1995). Light-regulated transcription. Annu. Rev. Plant Physiol. Plant Mol. Biol..

[50] Tatematsu K., Ward S., Leyser O., Kamiya Y., Nambara E. (2005). Identification of *cis*-elements that regulate gene expression during initiation of axillary bud outgrowth in *Arabidopsis*. Plant Physiol..

[51] Choi H., Hong J., Ha J., Kang J., Kim S.Y. (2000). ABFs, a family of ABA-responsive element binding factors. J. Biol. Chem..

[52] Ulmasov T., Hagen G., Guilfoyle T.J. (1999). Dimerization and DNA binding of auxin response factors. Plant J..

[53] Steudle E. (2000). Water uptake by roots: Effects of water deficit. J. Exp. Bot..

[54] Neuhäuser B., Dynowski M., Mayer M., Ludewig U. (2007). Regulation of NH_4_^+^ transport by essential cross talk between AMT monomers through the carboxyl tails. Plant Physiol..

[55] Vitart V., Baxter I., Doerner P., Harper J.F. (2001). Evidence for a role in growth and salt resistance of a plasma membrane H^+^-ATPase in the root endodermis. Plant J..

[56] Tamura K., Shimada T., Ono E., Tanaka Y., Nagatani A., Higashi S.I., Watanabe M., Nishimura M., Hara-Nishimura I. (2003). Why green fluorescent fusion proteins have not been observed in the vacuoles of higher plants. Plant J..

[57] Deng X.W., Quail P.H. (1999). Signalling in light-controlled development. Semin. Cell Dev. Biol..

[58] Buchanan-Wollaston V., Page T., Harrison E., Breeze E., Lim P.O., Nam H.G., Lin J.F., Wu S.H., Swidzinski J., Ishizaki K. (2005). Comparative transcriptome analysis reveals significant differences in gene expression and signalling pathways between developmental and dark/starvation-induced senescence in *Arabidopsis*. Plant J..

[59] Maurel C. (1997). Aquaporins and water permeability of plant membranes. Annu. Rev. Plant Physiol. Plant Mol. Biol..

[60] Naito S., Hirai M.Y., Chino M., Komeda Y. (1994). Expression of a soybean (*Glycine max* [L.] Merr.) seed storage protein gene in transgenic *Arabidopsis thaliana* and its response to nutritional stress and to abscisic acid mutations. Plant Physiol..

[61] (2007). EZ-C1 software, version 3.80.

[62] (2011). Photoshop CS5 extended software, version 12.1.

[63] Maeshima M. (1992). Characterization of the major integral protein of vacuolar membrane. Plant Physiol..

[64] (2004). MultiGauge software, version 3.0.

[65] Kaufmann S.H., Kellner U., Pound J.D. (1998). Erasure of western blots after autoradiographic or chemiluminescent detection. Immunochemical Protocols: Methods in Molecular Biology.

[66] Nakagawa T., Kurose T., Hino T., Tanaka K., Kawamukai M., Niwa Y., Toyooka K., Matsuoka K., Jinbo T., Kimura T. (2007). Development of series of gateway binary vectors, pGWBs, for realizing efficient construction of fusion genes for plant transformation. J. Biosci. Bioeng..

[67] Clough S.J., Bent A.F. (1998). Floral dip: A simplified method for *Agrobacterium*-mediated transformation of *Arabidopsis thaliana*. Plant J..

[68] Nagatani A., Reed J.W., Chory J. (1993). Isolation and initial characterization of *Arabidopsis* mutants that are deficient in phytochrome A. Plant Physiol..

[69] Suga S., Murai M., Kuwagata T., Maeshima M. (2003). Differences in aquaporin levels among cell types of radish and measurement of osmotic water permeability of individual protoplasts. Plant Cell Physiol..

