# Supplementary material for: Accumulation of TIP2;2 Aquaporin during Dark Adaptation Is Partially PhyA Dependent in Roots of Arabidopsis Seedlings
Source: Plants (Basel). 2014 Mar 5;3(1):177–95. doi: 10.3390/plants3010177 (PMC4844315; doi:10.3390/plants3010177)
Supplement: Supplementary File 1 [file plants-03-00177-s001.zip › plants-45206-supplement-fial/plants-45206-final-supplementary materials.docx]

Supplementary Materials

**Figure S1.** Transgenic *Arabidopsis* expressing a *TIP2;2-GFP* fusion gene. (**a**) Schematic diagram showing the *TIP2;2-GFP* fusion construct. Arrowheads indicate the primer binding sites used for RT-PCR. (**b**) RT-PCR amplification of total RNA isolated from roots of wild-type (Col) plants and two independent *TIP2;2-GFP* transgenic lines, 46-5 and 40.
(**c**) Immunoblotting of TIP2;2-GFP using root microsomal fractions. Col, Columbia (wild type); 46-5, TIP2;2-GFP/Col 46-5. (**d**,**e**) Subcellular localization of TIP2;2-GFP in root cells of light-grown Col (d: upper panels, root cells; lower panels, a protoplast), L*er* (e, left panel) and *phyA* (e, right panel) plants. GFP fluorescence was observed in a membrane-like structure in root cells of all lines. As a typical pattern, the membrane was dented at nuclei or other small organelles, indicating that the fluorescence is not from the plasma membrane. In protoplasts, fluorescence was also observed mainly on the tonoplast not on the plasma membrane. CBB, Coomassie brilliant blue. Bars, 20 µm.


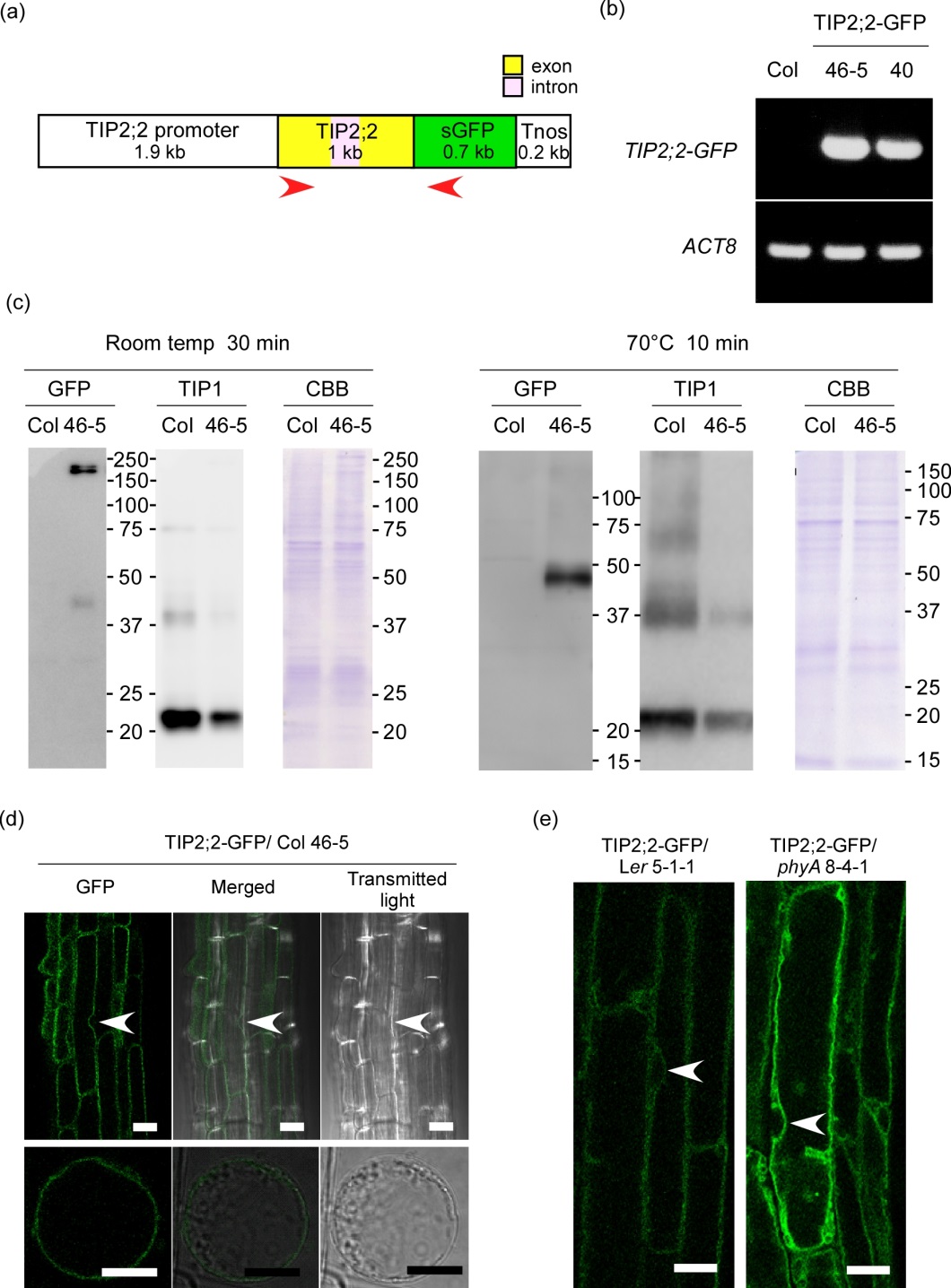


**Figure S2.** Measurement of the fluorescent intensity. (**a**) Representative image for measurement of fluorescent intensity. Twenty lines were drawn on a root and the fluorescent intensity on each line was measured. (**b**) A line graph of the fluorescent intensity on the red line at the upper side of (a). The maximum value of the fluorescent intensity on each line was measured. On each image, the mean of maximum values on twenty lines was calculated.


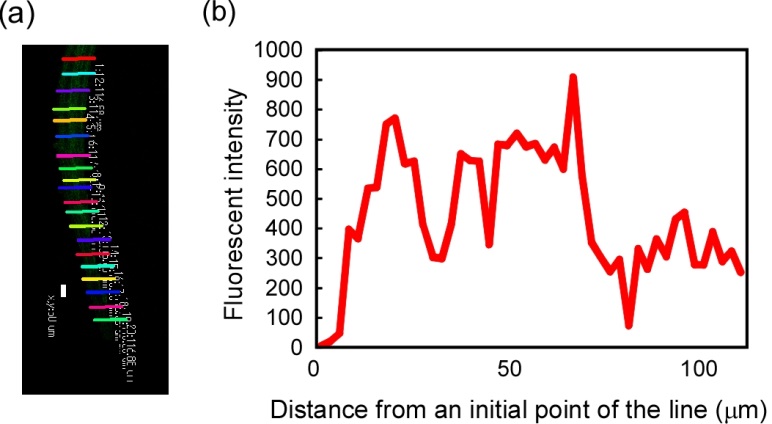


**Table S2.** List of primers used for PCR and sequencing.

| **Name** | **Nucleotide sequence** | **Number of bases** |
| --- | --- | --- |
| TIP2;2-1190F | 5’- CAC CGG CCG TCA GAC CTC AAC TGT TA -3’ | 26 |
| TIP2;2-3822F | 5’- CTC GCC GAC CAC TAC CAG CAG AA -3’ | 23 |
| TIP2;2-4035R | 5’- AGG GTA GCT TTC TGT GGT GGG AGC T -3’ | 25 |
| TIP2;2-GFP745F | 5’- GCC GCT TAA TCC ATA GTG ACA CG-3’ | 23 |
| TIP2;2-GFP1510F | 5’- AGC GGT AGT GGG TGG AAA AAC AT-3’ | 23 |
| TIP2;2-GFP2371R | 5’- TGC GAT AAA TTC AGA CAA GTA AG-3’ | 23 |
| TIP2;2-GFP2540F | 5’- CAT CTC CGG CGG ACA CCT TAA CC-3’ | 23 |
| TIP2;2-GFP3295F | 5’- GCA AGG GCG AGG AGC TGT TCA CC-3’ | 23 |
| TIP2;2-GFP4070R | 5’- TCG CAA GAC CGG CAA CAG GAT TC-3’ | 23 |
| TIP2;2cDNA-1F | 5'- ATG GTG AAG ATT GAG ATA GGA AG -3' | 23 |
| TIP2;2-sGFP-976R | 5'- AGG TGA AGG TGG TCA CGA G -3' | 19 |
| TIP2;2-sGFP-1226R | 5'- CGT TGT GGC TGT TGT AGT TGT -3' | 21 |
| ACT8F | 5'-ATG AAG ATT AAG GTC GTG GC -3' | 20 |
| ACT8R | 5'-TCC GAG TTT GAA GAG GCT AC -3' | 20 |
